# Different effects of the probe summarization algorithms PLIER and RMA on high-level analysis of Affymetrix exon arrays

**DOI:** 10.1186/1471-2105-11-211

**Published:** 2010-04-28

**Authors:** Yi Qu, Fei He, Yuchen Chen

**Affiliations:** 1 National Engineering Center for Biochip at Shanghai, Libing Rd. 151, Shanghai, 201203, China

## Abstract

**Background:**

Alternative splicing is an important mechanism that increases protein diversity and functionality in higher eukaryotes. Affymetrix exon arrays are a commercialized platform used to detect alternative splicing on a genome-wide scale. Two probe summarization algorithms, PLIER (Probe Logarithmic Intensity Error) and RMA (Robust Multichip Average), are commonly used to compute gene-level and exon-level expression values. However, a systematic comparison of these two algorithms on their effects on high-level analysis of the arrays has not yet been reported.

**Results:**

In this study, we showed that PLIER summarization led to over-estimation of gene-level expression changes, relative to exon-level expression changes, in two-group comparisons. Consequently, it led to detection of substantially more skipped exons on up-regulated genes, as well as substantially more included (i.e., non-skipped) exons on down-regulated genes. In contrast, this bias was not observed for RMA-summarized data. By using a published human tissue dataset, we compared the tissue-specific expression and splicing detected by Affymetrix exon arrays with those detected based on expressed sequence databases. We found the tendency of PLIER was not supported by the expressed sequence data.

**Conclusion:**

We showed that the tendency of PLIER in detection of alternative splicing is likely caused by a technical bias in the approach, rather than a biological bias. Moreover, we observed abnormal summarization results when using the PLIER algorithm, indicating that mathematical problems, such as numerical instability, may affect PLIER performance.

## Background

Alternative splicing (AS) contributes greatly to protein diversity throughout the evolution of complex organisms. According to Johnson et al. [1], about 70% of human multi-exon genes are predicted to have more than one isoform. Changes in the relative expression levels of the various isoforms may have significant biological implications (for example [2]). Genome-wide surveys of AS events have only become practical in recent years, largely due to the development of microarray technology. The Affymetrix exon array is one of the microarray platforms available for this purpose. It has been applied in a number of research areas [3-5], especially in cancer studies [6-8]. While traditional gene expression arrays target each transcript near the 3' end, Affymetrix Exon arrays target individual exons in the gene, thus enabling both gene-level expression analysis and detection of AS.

The Affymetrix GeneChip Human Exon 1.0 ST array has an extremely high probe density. The platform contains over 1.4 million probesets, each of which contains four perfect match (PM) probes that cover over 1 million exons. While this design has added new capacity to the microarray platform, it also poses new challenges in data analysis. First of all, gene-level and exon-level expression values need to be estimated using probe signals. This process is called summarization and several algorithms have been proposed for this purpose (for example [9]). The most commonly used summarization algorithms are RMA and PLIER, both of which are implemented in the Expression Console software provided by Affymetrix.

Both of the two algorithms implement a multiplicative error model. While RMA assumes that the error is proportional to the normalized and background-adjusted probe intensity, PLIER assumes that the error is proportional to the PM intensity without background correction. Define *t *as a target response which represents the abundance of the target mRNA, and *f *a feature response which represents the affinity of the probe. The model of RMA (on log scale) is specified as(1)

where *i *= 1,... *I *represents different arrays and *j *= 1,..., *J *represents different probes. *e*_*ij *_is an error term which follows the log-normal distribution. The model of PLIER is(2)

where *BKG*_*ij *_is the background value specific for array *i *and probe *j*.

Different approaches are taken to fit the models. RMA log-transforms the PM intensities, and uses median polish to obtain the robust estimates of log(*t*_*i*_) and log(*f*_*j*_). In contrast, PLIER algorithm works on the PM intensities directly without log-transformation. It defines *r*_*ij *_= log(*e*_*ij*_). In order to down-weigh outliner probes with large absolute values of *r*_*ij*_, the loss function is specified as , where *z *is a tuning constant for robustness. Newton's method is applied to find the values of *f *and *t *that minimize the loss function. The IterPLIER method, which is an extension of PLIER algorithm, generates gene-level signals based on consecutive exons [10,11]. A systematic comparison of PLIER and RMA summarization has not been reported. In this study, by using two public datasets, we found that IterPLIER and RMA derived different gene-level estimates from the same probe signals. Highly expressed probesets made more contribution to the gene-level signal in IterPLIER, compare to RMA.

Identification of differential splicing is also a challenge in microarray analysis. Changes in exon-level expression can be caused by two factors: differential splicing and differential gene expression. To detect differential splicing, the effect of differential gene expression must be removed. A commonly used strategy to achieve this is to calculate a NI (normalized index) for each exon, which is denoted as the ratio of the exon-level signal to the gene-level signal [12]. NI represents the exon inclusion rate and can be used in statistical testing to detect differential splicing between sample groups. This strategy eliminates gene-level expression in a simple manner. However, one possible disadvantage of this method is that it relies heavily on correct estimation of gene-level expression. When comparing PLIER and RMA, we discovered that the two methods behaved differently in detection of alternatively spliced exons and we found that a major cause for this phenomenon was the difference in estimation of gene expression between the two methods.

To assess the ability of PLIER and RMA to detect AS, a relatively large number of validated AS events are required. RT-PCR is often performed to verify microarray results, but large-scale validation of exon array results with RT-PCR can be very laborious and impractical. In this study, we proposed a different strategy. A large amount of data on expressed sequences have been collected for various human tissues and are available in public sequence databases (Refseq, dbEST, etc.). Noh et al. [13] previously used these data to identify tissue-specific expression and splicing. Their results are summarized in the TISA database. Since human tissues are expected to have good homogeneity, we compared a published human tissue panel dataset of Affymetrix exon arrays with these sequence analysis results. We measured the level of consistency between the two platforms and tested whether the tendency of PLIER or RMA to detect AS was supported by the sequence data.

## Methods

### Colon cancer dataset

The colon cancer dataset is available from the Affymetrix website [6]. Briefly, 10 matched pairs of human colon primary tumor and adjacent normal tissues were assayed on Affymetrix Human Exon 1.0 ST arrays. Only genes and exons with core annotation were considered in this study (the terms "exon" and "probeset" and the terms "gene" and "transcript" are used interchangeably hereafter). Patient 3 was removed since he was identified as an outlier by PCA analysis [6].

### Human tissue dataset

The human tissue panel dataset is also available from the Affymetrix website [14]. This dataset contains 11 tissues: breast, cerebellum, heart, kidney, liver, muscle, spleen, pancreas, prostate, testis and thyroid. Each tissue was assayed on Affymetrix Human Exon 1.0 ST arrays in three biological replicates. Only genes and exons with core annotation were used in this study.

### Data acquisition

Gene-level and exon-level signals were generated from CEL files with Expression Console v1.1 using the PLIER and RMA algorithms. In this paper, "PLIER summarization" refers to gene-level and exon-level signals derived with the iterPLIER and PLIER algorithms, respectively; while "RMA summarization" refers to both gene-level and exon-level signals derived with the RMA algorithm. RMA-generated signals were reported on a log_2 _scale, while PLIER-generated signals were reported on a linear scale. As recommended in [10], a value of 16 was added to the gene- and exon-level PLIER signals prior to log transformation. Unless otherwise specified, gene- and exon- level signals mentioned hereafter are assumed to be presented on a log_2 _scale.

### Data filtering

To reduce the false positive rate when comparing cancer vs. normal samples in the colon cancer dataset and when identifying tissue specificity in the human tissue dataset, we filtered probesets according to the suggestions in [6] and [12]. For the colon cancer dataset, we defined a probeset as present in a group if its DABG (detection above background) p-value < 0.05 in at least 50% of the samples in the group. We defined a transcript as present in a group if at least 50% of the core probesets of the transcript were present in the group. We retained probesets present in either of the two groups and transcripts present in both groups. Probesets with cross-hybridization type other than 1 (i.e., not all probes uniquely match the targeted exon) were also removed. Moreover, we kept only the genes with IterPLIER signal > 70 in order to increase the true positive rate (according to [6]).

For the human tissue dataset, similar filtering steps were performed. First, we defined a probeset as present in a tissue if its DABG p < 0.05 in at least 2 samples (out of 3) of that tissue. Since there are a total of 11 tissues in the dataset, we filtered for: (1) probesets present in either the test tissue or five of the other tissues; (2) genes present both in the test tissue and in five of the other tissues; (3) probesets with type 1 cross-hybridization. In addition, we retained only genes for which the mean expression ranked in the top 50% for both IterPLIER and RMA, since genes with low expression levels may associate with higher false positive rates in the detection of AS [12].

### Data analysis

As mentioned before, NI was calculated for each exon and used in statistical tests. For exon *i *in gene *j*, NI is denoted as (on log scale)(3)

where *E*_*i *_and *G*_*j *_are the expression values of exon *i *and gene *j*, respectively.

NI represents the inclusion rate of an exon in a sample, while the splicing index, SI, measures the difference in NI between two samples. For exon *i *in gene *j*, when comparing samples A and B, SI is denoted as (on log scale)(4)

For the colon cancer dataset, paired t-tests on gene-level signals were used to detect differential expression between normal and cancer samples. Paired t-tests on NI were used to detect differential splicing between the two groups. For the human tissue dataset, two sample t-tests were used on gene-level signals to detect tissue-specific expression (one tissue vs. all the others). Two sample t-tests were used on NI to detect tissue-specific splicing (one tissue vs. all the others).

### Comparison of the human tissue dataset with the TIssue-Specific Alternative splicing (TISA) database

TISA data was obtained from the website http://tisa.kribb.re.kr/AGC. Genes and exons in the database were mapped to transcripts and probesets on the exon array based on physical position. For each tissue in the TISA database, we counted the number of exons located on tissue-specific genes and with reported tissue-specific splicing. Then we conducted the chi-square test to determine whether the ratio of relatively skipped exons to relatively included exons was different from 1:1 to see if there is an enrichment of skipped exons on the tissue-specific genes. Tissue-specific expression and splicing events detected using the human tissue dataset were compared to the TISA database (see Additional file 1 for details).

## Results and discussion

### Comparison of gene-level estimation using PLIER and RMA

**Figure 1:**
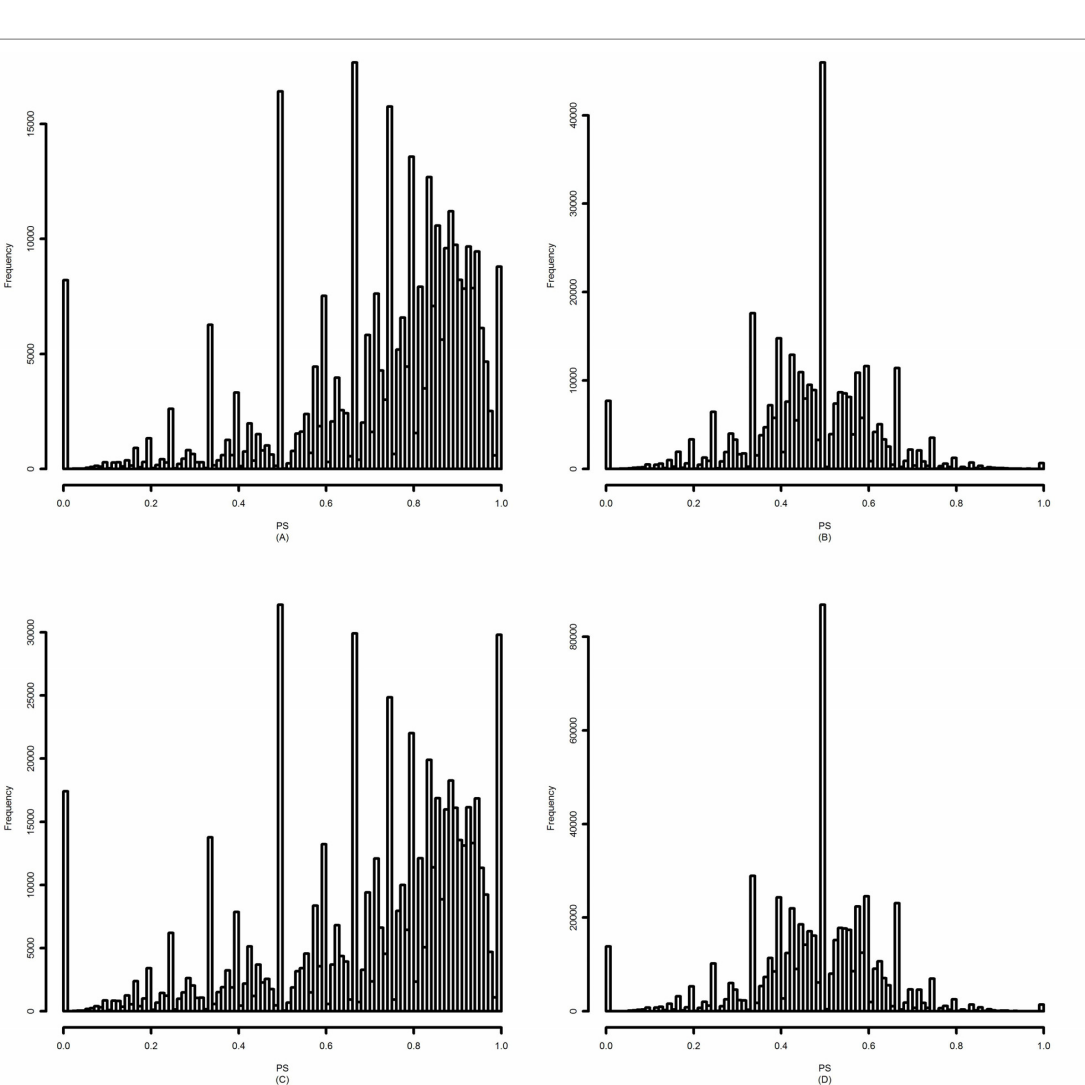
**Histogram of PS**. (A) Colon cancer dataset, PLIER summarization (mean = 0.725, std = 0.22) (B) Colon cancer dataset, RMA summarization (mean = 0.465, std = 0.148) (C) Human tissue dataset, PLIER summarization (mean = 0.702, std = 0.244) (D) Human tissue dataset, RMA summarization (mean = 0.479, std = 0.148)

Gene-level correction is essential for detection of AS using NI-based methods. To compare PLIER and RMA, we first compared the gene-level estimations derived by the two methods. We defined a quantity, named PS, as was the proportion of probesets whose signals were smaller than the corresponding gene-level signal in a specific sample. PS was calculated for all the core transcripts in all samples. As illustrated in Figure 1, the distribution of PS for RMA-summarized data is roughly symmetrical and centered at 0.5. In contrast, the distribution of PS for PLIER-summarized data is right-skewed. Although the PLIER and RMA algorithms did not explicitly assign weights to probesets in gene-level estimation, this result shows that PLIER tends to "weigh" probesets with higher expression values more heavily than RMA.

In microarray analysis, usually the fold change of gene expression between arrays, rather than the gene expression value itself, is of interest. In perfect case where the expression levels of exons change exactly proportionally across arrays, the "weighing scheme" may not affect the estimation of gene-level expression changes, however, as we will shown in the next section, in real situation, the two methods did result in different estimation of the fold change of gene expression, which lead to detection of different AS events with NI-based methods.

### Comparison of AS detection using PLIER and RMA

The two datasets were used to compare exons identified as alternatively spliced with either PLIER or RMA. For the colon cancer dataset, we compared cancer vs. normal samples and for the human tissue dataset, we compared cerebellum vs. non-cerebellum tissues, due to the high number of reported tissue specific splicing events in the brain.

After filtering, a total of 54,908 core probesets located in 3,552 core transcripts were retained in the colon cancer dataset. In the human tissue dataset, for the comparison of cerebellum vs. non-cerebellum tissues, 111,703 core probesets located in 7,686 core transcripts were kept for further analysis.

Paired t-tests and Welch tests were applied on NI to identify alternatively spliced exons in the colon cancer dataset and in the human tissue dataset, respectively. We observed different tendencies in detection of AS between PLIER and RMA. As mentioned before, SI represents the difference in NI for a given exon between two samples. Because of the different experimental designs of the two datasets (paired and unpaired), we defined SI for the two datasets separately. For the colon cancer dataset, we define(3a)

where *SI *represents the difference in NI for exon *i *in gene *j *in the k_th _sample pair, and Δ*G *represents the difference in expression levels of gene *j *for the k_th _sample pair.

For the human tissue dataset, we define(5)

where *SI*_*ij *_and Δ*G*_*j *_represent the mean difference in inclusion rates of exon *i *and in expression levels of gene *j *between the cerebellum vs. non-cerebellum tissues, respectively.

In both datasets, we found that SI was strongly negatively correlated with Δ*G *for PLIER-summarized data, while SI and Δ*G *were correlated to a much lesser extent (though the correlation was also significant, p < 0.01) for RMA-summarized data. Person and Spearmen correlations between *SI*_*i*, *j*, *k *_and Δ*G*_*j*, *k *_in the colon cancer dataset were as large as -0.492 and -0.456, respectively, for the PLIER-summarized data. In contrast, the correlations were 0.048 and 0.045 for the RMA-summarized data. Similarly, in the human tissue dataset, the Person and Spearmen correlations between *SI*_*i*, *j *_and Δ*G*_*j *_were -0.58 and -0.60 when using PLIER, and -0.021 and 0.055 when using RMA, respectively.

**Figure 2:**
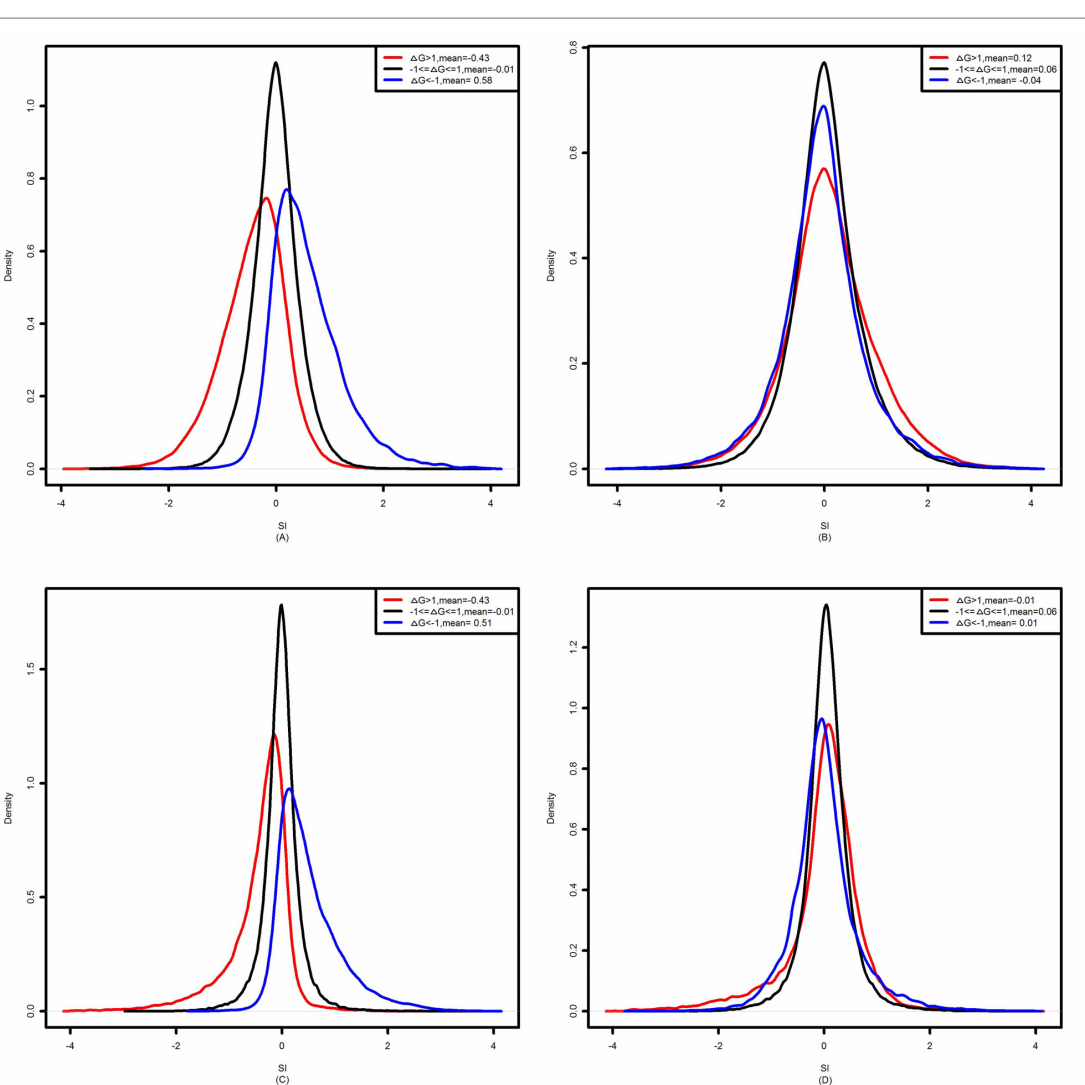
**Distribution of SI**. (A) SI calculated with PLIER in the colon cancer dataset (B) SI calculated with RMA in the colon cancer dataset (C) SI calculated with PLIER in the human tissue dataset (D) SI calculated with RMA in the human tissue dataset.

The negative correlations indicate that PLIER summarization leads to detection of more included exons on down-regulated genes, as well as more skipped exons on up-regulated genes. This is illustrated in Figure 2 and in Table 1. Figure 2 shows a plot of the density of SI in three cases: (1) Δ*G *> 1, (2) -1 ≤ Δ*G *≤ 1, (3) Δ*G *< -1. For PLIER in both datasets when Δ*G *> 1, SI is relatively negatively distributed; when -1 ≤ Δ*G *≤ 1, the distribution of SI centers at zero; and when Δ*G *< -1, SI is relatively positively distributed. However, the distributions of SI in the three cases for RMA are all centered around zero.

**Table 1:** Detection of alternatively spliced exons on differentially expressed genes: (A) Human tissue dataset, (B) Colon cancer dataset

Method	Significance level of AS	# of significant probesets	# of significant probesets located on differentially expressed genes^a^(1)	# of probesets in (1) and with mean gene expression difference (2)	# of probesets in (2) and with mean NI difference
PLIER	0.001	13555	10737	>0 6513	>0 559 (8.6%)
					<0 5954 (91.4%)
				<0 4224	>0 4086 (96.7%)
					<0 138 (3.3%)
	0.05	37746	26652	>0 16456	>0 1736 (10.5%)
					<0 14729 (89.5%)
				<0 10187	>0 9641 (94.6%)
					<0 573 (5.4%)
					
RMA	0.001	9433	6033	>0 4381	>0 2485 (56.7%)
					<0 1896 (43.3%)
				<0 1652	>0 1067 (64.6%)
					<0 585 (35.4%)
	0.05	31238	18628	>0 12318	>0 7295 (59.2%)
					<0 5023 (40.8%)
				<0 6310	>0 3575 (56.7%)
					<0 2735 (43.3%)
**Method**	**Significance level of AS**	**# of significant probesets**	**# of significant probesets located on differentially expressed genes^b^(1)**	**# of probesets in (1) and with mean gene expression difference (2)**	**# of probesets in (2) and with and mean NI difference**

PLIER	0.001	330	220	>0 200	>0 3 (1.5%)

					<0 197 (98.5%)

				<0 20	>0 20 (100%)

					<0 0 (0%)

	0.05	8359	3283	>0 2830	>0 134 (4.7%)

					<0 2696 (95.3%)

				<0 453	>0 436 (96.2%)

					<0 17 (3.8%)

					

RMA	0.001	230	107	>0 105	>0 35 (33.3%)

					<0 70 (66.7%)

				<0 2	>0 0(0%)

					<0 2 (100%)

	0.05	7233	2602	>0 2482	>0 1372 (55.3%)

					<0 1110 (44.7%)

				<0 120	>0 60 (50%)

					<0 60 (50%)

a: significance level for differential gene expression was p < 0.001

b: Due to the relatively small number of differentially expressed genes, the significance level for differential gene expression here was set to p < 0.01

Table 1 shows the number of probesets that were identified as having significant AS and were located on up- or down-regulated genes. Two significance levels of AS were considered, p < 0.001 and p < 0.05. For PLIER-summarized data, at least 89.5% of the significant probesets detected on up-regulated genes were relatively skipped (with SI < 0) and at least 94.6% of the significant probesets detected on down-regulated genes were relatively included (with SI > 0). In contrast, for RMA summarized data, substantially more balanced numbers of skipped and included probesets were detected on both up- and down-regulated genes (the ratio of numbers of these two kinds of probesets ranged between 3:7 and 7:3 in all but one case, where the total number of probesets under consideration was only 2). (Table 1)

**Figure 3:**
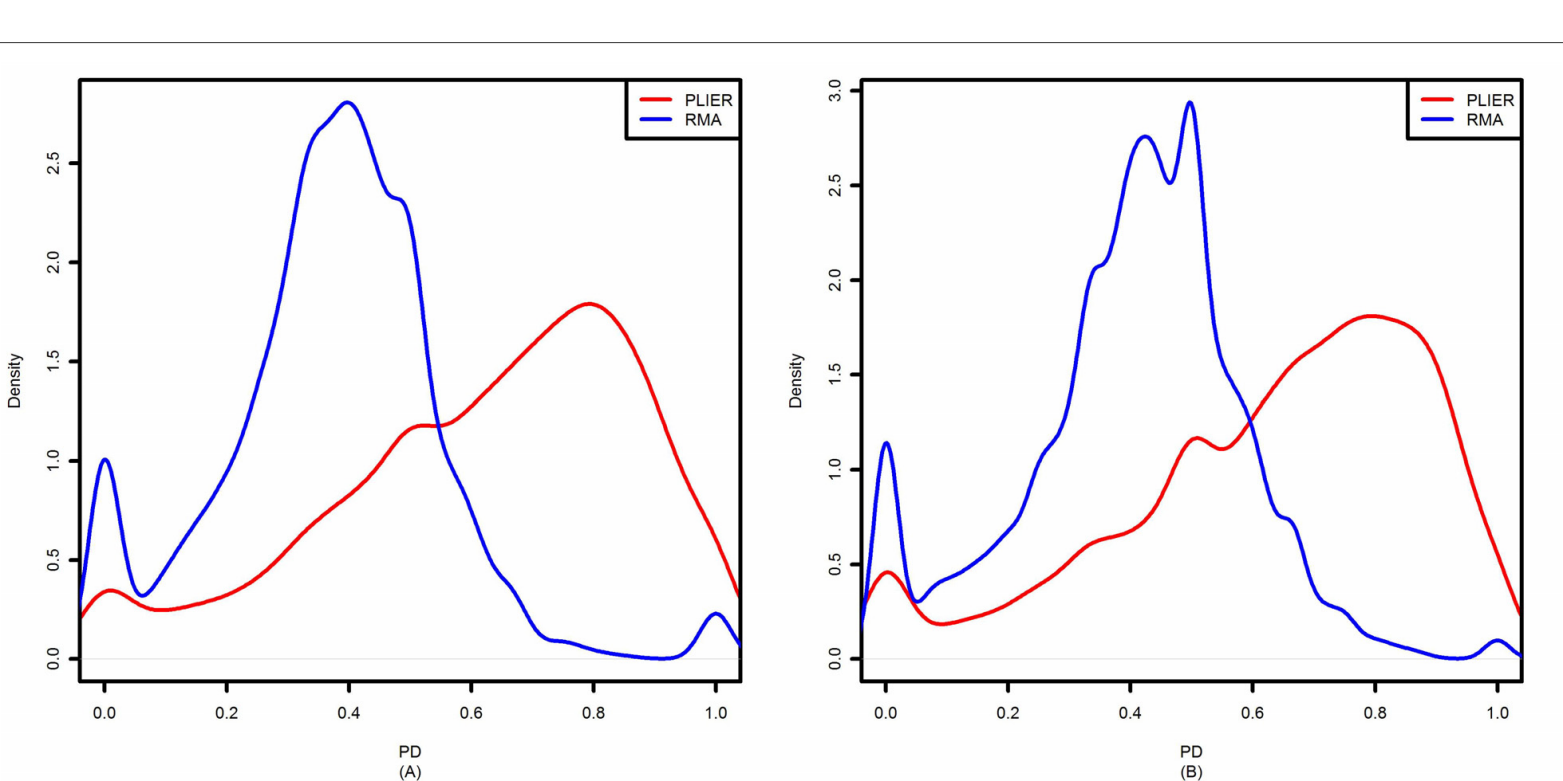
**Distribution of PD**. (A) Colon cancer dataset (B) Human tissue dataset.

Another point that can be inferred from the negative correlation between SI and Δ*G *is that PLIER leads to over-estimation of gene-level expression changes between the sample groups, relative to the exon-level expression changes. Since by definition,

so SI actually compares the magnitude of exon-level expression changes with the corresponding gene-level expression change between samples. Over-estimation of gene-level expression change, (i.e., |*G*_*j*, *A *_- *G*_*j*, *B*_| > |*E*_*j*, *A *_- *E*_*i*, *B*_| for most of the exons in gene *j*), leads to the negative correlation between SI and Δ*G*. Figure 3 shows the distribution of a quantity, PD, which is the proportion of probesets whose absolute mean expression difference is smaller than the corresponding absolute mean gene expression difference between the sample groups under consideration (for about 95% of all the exons, the exon-level expression change and corresponding gene expression change were either of the same sign or close to zero). This figure clearly shows the tendency of PLIER to over-estimate gene-level expression differences, relative to exon-level expression differences.

**Figure 4:**
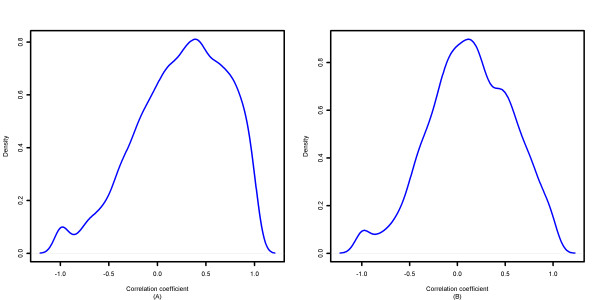
**Distribution of Pearson correlation coefficients**. The Pearson correlation coefficient between the mean exon expression level and the absolute difference in exon expression (between the two sample groups under consideration) was computed using PLIER-summarized data for each gene. (A) Human tissue dataset (B) Colon cancer dataset

We demonstrated that PLIER tended to "weigh" highly expressed probesets more heavily and over-estimate gene expression differences. These two observations can be linked together if probesets with higher expression are likely to be associated with larger exon-level expression differences. As shown in Figure 4, we found that mean exon expression actually was moderately positively correlated with the magnitude of exon expression difference between groups for PLIER-summarized data. The distribution of Pearson correlation coefficients between mean exon expression and the absolute value of exon expression difference was right-skewed with mean = 0.246 and std = 0.462 for the human tissue dataset and mean = 0.13 and std = 0.431 for the colon cancer dataset (t-test for μ = 0 p < 10^-5 ^in both datasets). This observation helps to explain what we have observed for PLIER gene-level estimation.

One possible reason for the observed difference between PLIER and RMA summarization is that, the multiplicative error model may not be completely hold for probes with low intensities. As it is generally thought that low intensity features are likely to be associated with larger coefficients of variation, lowly expressed probesets are typically filtered out in microarray analysis. So to minimize the loss function , PLIER probably down-weighs features with low intensities. The iterPLIER algorithm may further strengthen the tendency of "selecting against" low intensity features, since it first computes the gene-level expression using all the probes with PLIER, and then iteratively selects a subset of probes whose signal varies in a similar pattern as the predicted gene-level expression to redo the calculation. Another point to be considered is that, as suggested by the moderately positive correlation between the average exon-level expression and expression difference between sample groups, the assumption that *f*_*j *_is independent of signal intensity is probably not completely hold either, which may also have an influence on gene-level estimation.

### Assessment of the enrichment level of relatively skipped exons on up-regulated genes using the TISA database

Noh et al. used mRNA and EST sequences from public databases to identify tissue-specific gene expression and splicing in humans and mice. The work flow of their study was thoroughly described in [13] and their results were summarized in the TISA database. Briefly, the expressed sequences were retrieved from GenBank and dbEST, classified based on tissue origin and mapped to genomic sequences to establish gene units and reconstruct transcripts. Statistical tests were used to identify tissue-specific gene expression and splicing. For humans, 26,143 genes were reconstructed, of which 13,015 were determined to have more than one isoform and were located on autosomes. These 13,015 genes were used in this study. A total of 200,619 exons were listed for these genes, 63.8% of which were mapped to core probesets on the exon array. Since human tissue data are expected to have good homogeneity, we compared the human tissue dataset of exon arrays with the TISA database in detection of tissue-specific expression and splicing. For detection of tissue specific splicing using PLIER-summarized exon array data, we found that, besides the cerebellum, all other tissues showed remarkable enrichment (range from 85% to 96%, see Table 2) of relatively skipped exons on tissue-specific genes (i.e., genes that were significantly up-regulated in the tissue). If this observation reflects the true situation, then the same tendency should be observed in the TISA database. (Table 2)

**Table 2:** Enrichment of skipped exons on tissue-specific genes in the human tissue dataset

tissue	Pearson correlation between SI and Δ*G*	Spearman correlation between SI and Δ*G*	enrichment of skipped probesets (p < 0.001) on tissue-specific genes	enrichment of skipped probesets (p < 0.05) on tissue-specific genes
breast	-0.47	-0.45	95.8%(1321/1379)	92.7%(4065/4385)
cerebellum	-0.58	-0.60	91.4%(5954/6513)	89.4%(14729/16465)
heart	-0.47	-0.46	90.8%(1437/1582)	87.4%(3898/4458)
kidney	-0.47	-0.43	92.4%(1004/1086)	91.4% (3035/3322)
liver	-0.52	-0.52	91.9% (2574/2802)	85.3% (6595/7734)
muscle	-0.49	-0.49	90.5% (1975/2183)	86.2%(5430/6296)
pancreas	-0.43	-0.39	93.6%(508/543)	89.3% (1576/1764)
prostate	-0.44	-0.40	93.8%(609/649)	92.1% (1721/1868)
spleen	-0.49	-0.47	94.7% (2680/2830)	90.2% (6941/7692)
testes	-0.53	-0.55	96.3% (3659/3801)	94.4% (10059/10645)
thyroid	-0.45	-0.41	92.7%(939/1013)	88.2% (2574/2919)

The TISA database contains 46 tissues. A total of 4,527 exons were found to be involved in the 3,695 tissue-specific splicing events (with p < 0.05), while 1,753 of these exons were located on genes that were specifically expressed in the same tissue (an exon may be counted multiple times if it is involved in more than one tissue-specific splicing event). Nine exons with inconsistent tissue specificity (i.e., presents in both isoform A, which was reported to be more enriched in a given tissue, and isoform B, which was reported to be less enriched in the same tissue) were removed, and the remaining 1,744 exons were kept for further analysis.

Several factors may affect the comparability of the human tissue dataset and the TISA database: (1) splicing forms. The TISA database contains 8 splicing forms, of which 3 splicing forms, 'cassette exon', 'multiple cassette exon' and 'mutual exclusive exon' are most readily detectable by the exon array, while splicing forms such as 'intron retention' may not be detectable; (2) tissue content. The TISA database contains 46 tissues, while the human tissue dataset contains only 11 tissues. If only exactly matched tissue names are considered, 10 tissues (all tissues from the array dataset excluding breast) are present in both cases; (3) mapping between the two platforms. Only core probesets on the exon array were considered in this study. This may result in a bias in the mapping, since tissue-specific exons in the TISA database may be present in smaller number of mRNAs and thus may be less likely to be mapped to core probesets. By considering combinations of the 3 factors, we listed a total of 8 cases in Table 3. Significant enrichment of skipped exons on genes with tissue-specific expression was not observed in any of these cases, indicating that the tendency of PLIER to detect substantially more skipped exons in these genes was not supported by the TISA data. To date, there is no clear evidence or established theory supporting strong negative correlation between SI and Δ*G*. So we believe the tendency of PLIER to predict these events is due to technical bias, as opposed to a biological bias. (Table 3)

**Table 3:** Numbers of exons located on tissue-specific genes and with tissue-specific splicing, according to TISA database

	# exons with tissue-specific splicing and located on genes with tissue-specific expression (1)	# exons in (1) and were relatively included in that tissue	# exons in (1) and were relatively skipped in that tissue	Chi-square p-value
all	1744	877	867	0.865
3 splicing forms only	647	326	321	0.912
10 tissues only	706	383	323	0.11
3 splicing forms+10 tissues only	246	133	113	0.367
all exons mapped to core probesets	1061	518	543	0.572
3 splicing forms+ mapped to core probesets only	323	158	165	0.813
10 tissues +mapped to core probesets only	413	214	199	0.626
3 splicing forms+10 tissues+ mapped to core probesets only	128	71	57	0.381

### Comparison of tissue-specific gene expression and splicing events detected using the human tissue dataset with those reported in the TISA database

This study compared gene- and exon-level tissue specificity identified using the exon arrays with the TISA database to assess reliability of the exon array platform and the performance of PLIER and RMA. The mapping between the two platforms showed reasonable agreement in gene- and exon-sequence clustering. At the gene level, we observed significant consistency between the two platforms in detection of tissue-specific expression for both PLIER- and RMA-summarized data. RMA performing slightly better than PLIER in distinguishing "true positives" from "true negatives", where the tissue-specific expression reported in TISA were assumed to be true events. However, at the exon-level, the consistency between the two platforms was not significant, regardless of the summarization method. In addition, the difference between the two methods was not significant. Due to the lack of significant agreement between the TISA database and the tissue dataset, we did not reach a conclusion as to which method was better (see Additional file 1 for details).

### Possible numerical instability problem in PLIER summarization

**Figure 5:**
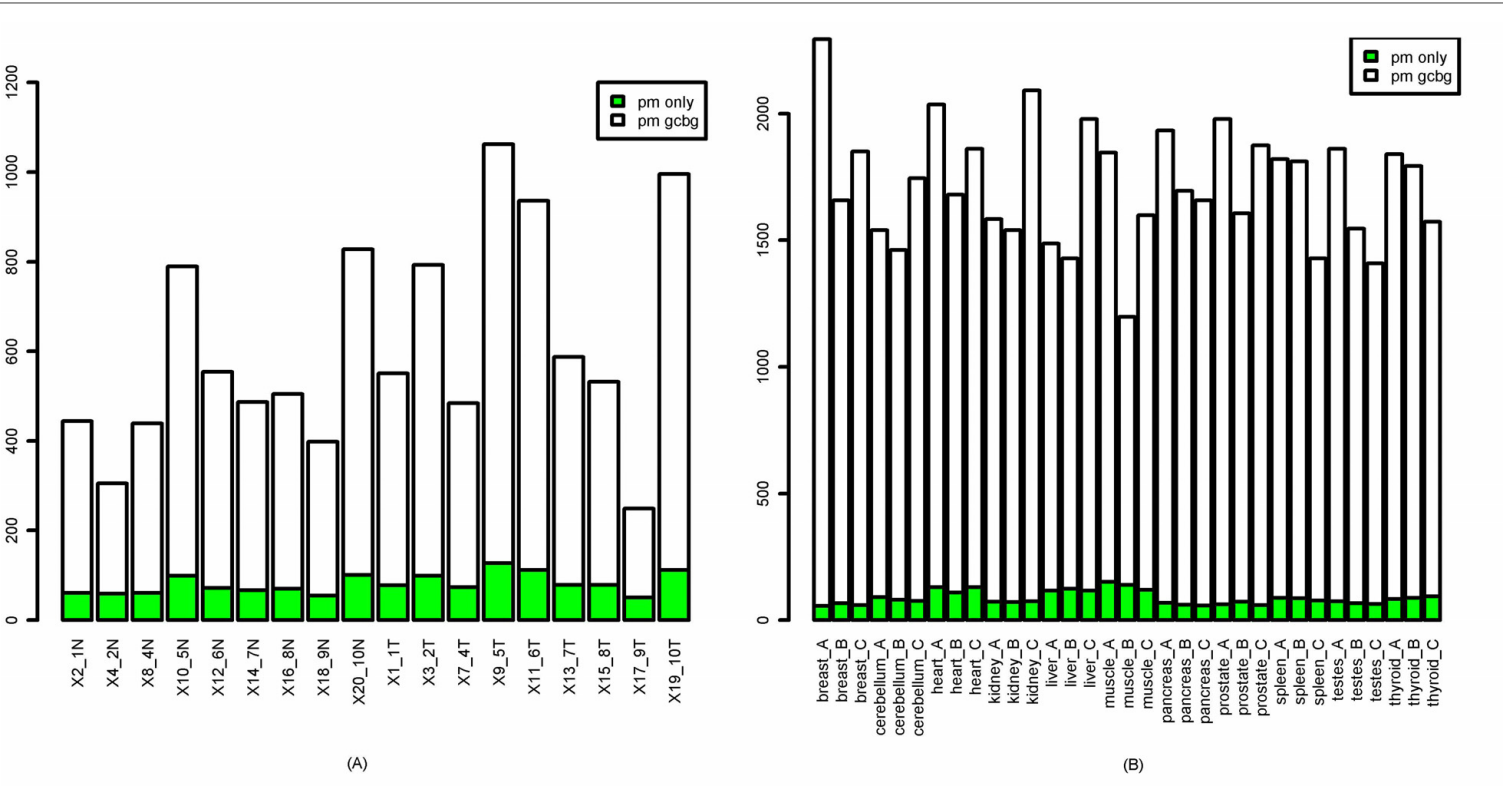
**The expression values of two genes calculated using the iterPLIER algorithm with different background-correction options**. The x-axis represents samples and the y-axis represents gene expression values (on a linear scale) (A) Transcript 2623139 in the colon cancer dataset (B) Transcript 3190762 in the human tissue dataset.

The Expression Console software offers two options for background correction for IterPLIER or PLIER, called 'pm' and 'pm-gcbg'. The 'pm' option uses the signals of PM probes directly (without background correction) for calculation of gene-level or exon-level expression, while the 'pm-gcbg' method corrects the PM signal by subtracting a GC-content specific background signal. Thus, we expected the gene-level or exon-level signals computed with the 'pm-gcbg' option to be slightly smaller than those computed with the 'pm' option. Although this was true for the vast majority of genes and exons, to our surprise, the expression values computed with the 'pm-gcbg' option for a small proportion of genes and exons were much greater than those computed with the 'pm' option. Figure 5 shows two typical genes displaying this behavior (with the mean expression calculated with 'pm-gcbg' at least 7 fold greater than the mean expression calculated with 'pm'), one from the colon cancer dataset, and one from the human tissue dataset. A similar phenomenon was also observed for exon-level signals (data not shown). The expression values shown in the plots are displayed on a linear scale.

Since the IterPLIER algorithm is based on PLIER, and PLIER relies on Newton's method to find the best solution for parameters, the observed phenomena may be due to numerical instability, which can cause the algorithm to be trapped in a local maximum, resulting in retrieval of an abnormal solution for the parameters. By using the APT software (Affymetrix Power Tools) and choosing different parameters for controlling the PLIER algorithm, similar problems can be avoided in some cases, but not in all (data not shown).

## Conclusion

In this study, we found that the two commonly used summarization algorithms, PLIER and RMA, behaved differently in detection of AS. Due to different gene-level estimation, PLIER showed a strong tendency to detect relatively skipped exons on up-regulated genes and relatively included exons on down-regulated genes, while this tendency was not observed when using RMA. To determine whether this tendency of PLIER represents a real biological situation, we used tissue-specific expression and splicing events that have been identified with sequence data and summarized in the TISA database as references. The TISA data did not show significant enrichment of skipped exons on genes with tissue-specific expression, a finding that did not support the tendency of PLIER. So we concluded that the observed tendency of PLIER is due to technical bias. We also compared the performance of RMA and PLIER in detection of AS by using tissue-specific splicing events in the TISA database as true positives. The consistency between the exon array data and the TISA database was low for both summarization methods, and the difference between the two methods was not significant. Given the observed bias of PLIER, this result may suggest that the efficacy of the RMA algorithm can be further improved as well. More sophisticated methods that incorporate sequence information or other characteristics of the probes may help to achieve more accurate estimation of gene- and exon-level expression [15].

## Abbreviations

AS: (Alternative Splicing); PLIER: (Probe Logarithmic Intensity Error); RMA: (Robust Multichip Average); DABG: (Detection above Background); NI: (Normalized Index); SI: (Splicing Index); TISA: (Tissue-specific Alternative splicing).

## Authors' contributions

The work presented here was carried out in collaboration between all authors. YC and YQ defined the research theme. YQ, YC and FH designed methods and experiments, analyzed the data, interpreted the results and wrote the paper. All authors have contributed to, seen and approved the manuscript.

## Supplementary Material

Additional file 1 **Comparison of the analysis results of the human tissue dataset and the TISA database**. Detailed description on how the tissue-specific genes and exons identified with the human tissue dataset were compared to those reported in TISA database in this study. Click here for file
