# Supplementary material for: Different effects of the probe summarization algorithms PLIER and RMA on high-level analysis of Affymetrix exon arrays
Source: BMC Bioinformatics. 2010 Apr 28;11:211. doi: 10.1186/1471-2105-11-211 (PMC2873539; doi:10.1186/1471-2105-11-211)
Supplement: Additional file 1 — Comparison of the analysis results of the human tissue dataset and the TISA database. Detailed description on how the tissue-specific genes and exons identified with the human tissue dataset were compared to those reported in TISA database in this study. [file 1471-2105-11-211-S1.PDF]

Comparison of the analysis results of the human tissue dataset and the TISA database

A. Mapping between the two platforms

For the TISA database, we considered 13,015 reconstructed genes that were reported to have more than one isoform and were on autosomes. There were a total of 200,619 exons listed for the 13,015 genes in the database (these genes and exons are denoted as AGC genes and AGC exons, respectively, where AGC stands for Animalia Gene Catalog). AGC exons were mapped to probesets on the exon array based on physical locations. If a probeset was located completely inside an AGC exon, we defined a mapping between the AGC exon and the probeset. We found that 128,059 AGC exons mapped to 218,935 core probesets (corresponding to 114,273 exons on the exon array). There were 113,448 (88.6%) AGC exons uniquely mapped to a single exon ID on the exon array, and 92,189 (80.7%) exon IDs on the exon array uniquely mapped to a single AGC exon. For gene-level consistency of sequence clustering, 10,410 (87.7%) AGC genes uniquely mapped to one transcript ID on the array, and 10,829 (89.5%) transcript IDs uniquely mapped to one AGC gene. Considering that not exactly the same sequence data were used in clustering and different approaches and criteria were applied (see [1] for detailed description for exon array annotations and transcript clustering), these results showed reasonable agreement between the two studies.

B. Comparison of the detection of tissue-specific gene expression

We compared the detection of tissue-specific expression using the sequence data and the exon arrays. Of the 13,015 genes in the TISA database, 8,484 were identified as displaying tissue-specific expression in at least one of the 46 tissues. The human tissue dataset includes 11 tissues. To avoid ambiguity when comparing the exon array data with the TISA database, we only considered exactly matched tissue names. Thus, ten tissues (all except breast from the tissue dataset) were kept for further analysis. In total, 4,725 tissue-specific expression events were reported in these tissues (with $p<0.001$). By including only one-to-one mapped AGC gene IDs and transcript IDs, we

obtained a list of 3,477 tissue-specific expression events involving 3,010 genes on the exon array (this gene set was denoted as the test set).

T-tests (one tissue vs. all the others) were performed to detect tissue-specific expression in PLIER- and RMA-summarized gene-level data. For each of the 10 tissues, we counted the number of genes that were both significantly up-regulated (with $p<0.001$ or $p<0.05$) according to the exon array data and reported as specifically expressed in that tissue in the TISA database. As shown in Fig. S1, at a significance level $p<0.001$, 1,908 (54.8%) and 1,701 (48.9%) of the 3,477 tissue-specific expression events were confirmed by the exon array data for PLIER and RMA, respectively. At a significance level $p<0.05$, the number of consistent tissue-specific expression events increased to 2,410 (69.0%) and 2,342 (67.4%), for PLIER and RMA, respectively.

Figure S1 Numbers of genes with consistent tissue-specific expression reported in the TISA database and detected with the exon array dataset. (A) $p<0.001$ (B) $p<0.05$

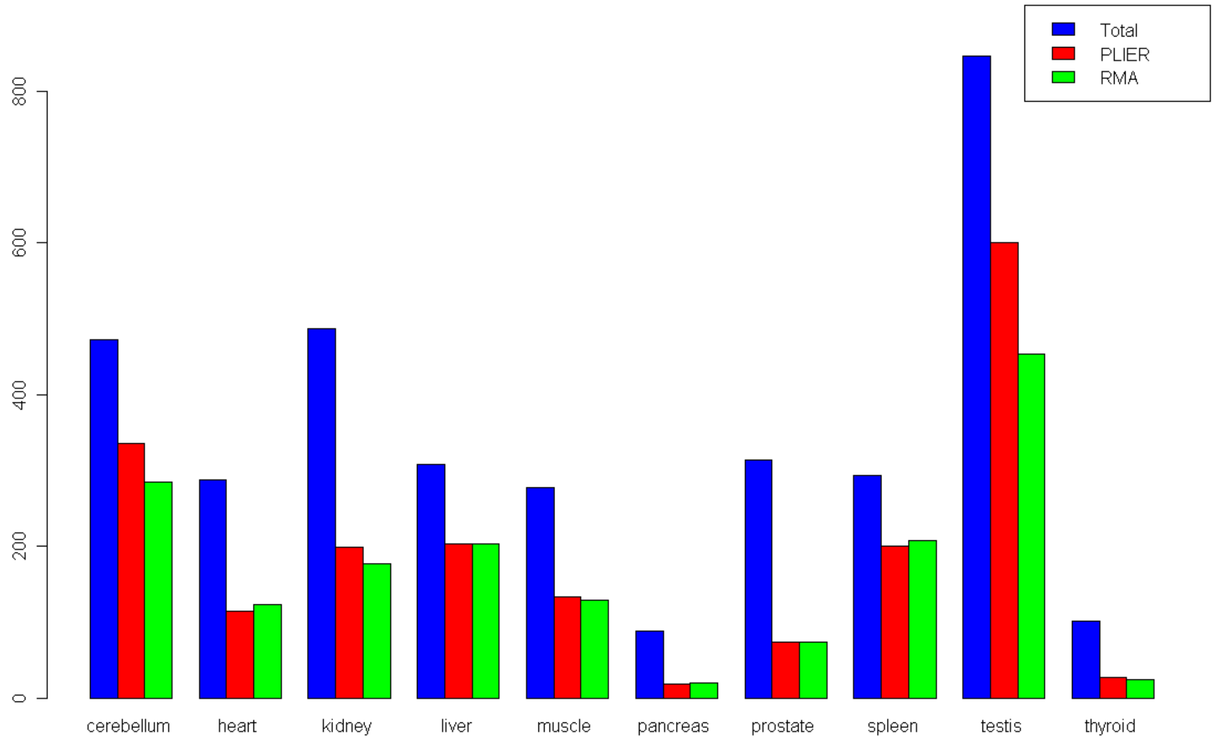
Total
PLIER
RMA
0
200
400
600
800
cerebellum
heart
kidney
liver
muscle
pancreas
prostate
spleen
testis
thyroid

(A)

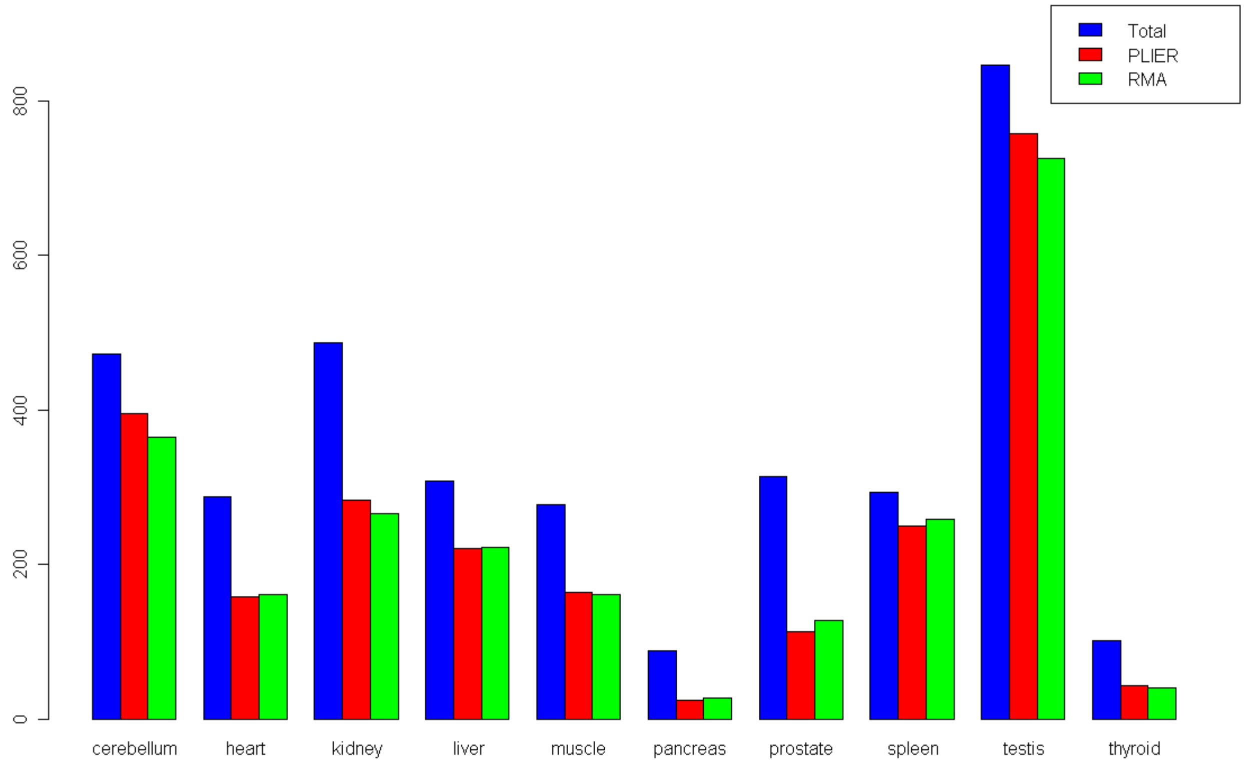
Total
PLIER
RMA
0
200
400
600
800
cerebellum
heart
kidney
liver
muscle
pancreas
prostate
spleen
testis
thyroid

(B)

To further compare the performance of PLIER and RMA, we considered both sensitivity and specificity. We defined a reference set, which was composed of genes without reported tissue-specific expression in any of the 46 tissues. For each tissue under consideration, we randomly sampled the same number of genes contained in the test set from the reference set. By using the test set as true positives, the randomly sampled genes from the reference set as true negatives, and the product of logged t-test p-value and the sign of ΔG as the classification variable, we created ROC (receiver operation characteristic) curves and calculated AUC (area under curve) values separately for PLIER- and RMA-summarized data (the resultant AUC values were denoted as AUC PLIER and AUC RMA, respectively). The random sampling experiment was repeated 100 times, and 100 AUC values were calculated for each summarization method and each tissue. We performed t-tests on the AUC values and Fisher's exact tests on the number of cases in which AUC PLIER was greater or smaller than AUC RMA (to see if the number of the two cases was 1:1). As shown in Table S1, at significance level $p<0.05$, the two tests gave the same result. RMA significantly outperformed PLIER in 7 out of the 10 tissues (cerebellum, heart, kidney, pancreas, prostate, spleen, and testis), and PLIER significantly outperformed RMA in 2 tissues (liver and thyroid). Among all the 1000 cases (10 tissues and 100 random sampling experiments per tissue), the values of AUC RMA were greater than AUC PLIER in 740 cases (Fisher's exact test p=7.74E-29). Based on these observations, we concluded that RMA performed better than PLIER in detection of tissue-specific expression. To determine whether the level of consistency between the exon array data and the TISA database was higher than random, we used random draws from the Uniform distribution as the classification variable to re-compute the AUC values (these resultant AUC values were denoted as AUC Uniform). In all the tissues, AUC PLIER and AUC RMA were significantly greater than AUC Uniform (t-test $p<0.001$), showing that the agreement between the two platforms was significant in detection of tissue-specific gene expression.

C. Comparison of the detection of tissue-specific splicing

A total of 3,695 tissue-specific AS events on autosomes were reported in the TISA database (with p<=0.05). Again, to avoid ambiguity, we only considered the 10 tissues mentioned above. A total of 1,171 AS events, which can be classified into eight forms, were reported in the 10 tissues. The eight forms include: 3’ splice site variation (10.8%), 5’ splice site variation (8.9%), cassette exon (53.3%), composite splice variation (3.2%), introns retention (9.3%), multiple cassette exon (10.4%), mutually exclusive exon (3.5%) and overlapping exon (0.4%). In this study, we only focused on three AS forms: cassette exon, multiple cassette exons, and mutually exclusive exon, since these AS forms should be detectable on the exon array, as long as the involved exons are covered by the core probesets. After the filtering, 789 AS events were retained for further analysis.

A total of 797 AGC exons and 570 AGC genes were found to be involved in the 789 AS events. The list of exons involved in more than one AS event in a tissue was reviewed manually and an exon with inconsistent inclusion patterns (i.e., be present both in isoform A, which was reported to be less enriched in a certain tissue, and in isoform B, which was reported to be more enriched in the same tissue) was removed. The remaining exons were mapped to probesets on the exon array. After the filtering, 379 AGC exons in 302 AGC genes mapped to 494 probesets. By excluding genes on the exon array which were not one-to-one mapped to AGC genes, we ultimately retained 388 probesets, corresponding to 264 AGC exons on 219 AGC genes. These probesets were denoted as the test set. A probeset may be counted multiple times if its corresponding AGC exon was reported to undergo AS in more than one of the 10 tissues. The test set contained 356 unique probesets on 263 exons and 219 transcripts on the exon array.

The number of probesets reported to be specifically spliced in each tissue is listed in Table S2. Two-sample t-tests based on NI were used to identify tissue-specific splicing (one tissue vs. all the other tissues). To compare tissue-specific splicing

detected with the exon array data with those splicing events reported in the TISA database, we considered two problems: (1) whether the probesets reported to be alternatively spliced in TISA were also significant in the exon array data, and (2) whether the exon inclusion/exclusion patterns (i.e., the sign of SI) detected with the exon array data agreed with those reported in the TISA database.

A reference set was created, composed of probesets which satisfied (1) the corresponding AGC exons were not reported to be alternatively spliced in any tissue, and (2) the probeset was located on one-to-one mapped genes that were involved in tissue -specific splicing. First, we compared the distribution of p-values in the test set with the distribution in the reference set. Five tissues with greater than 30 probesets in the test set were considered (cerebellum, kidney, liver, muscle, and testes). As we did for the gene-level comparison, for each of the five tissues, 100 randomly sampled datasets were generated from the reference set. Logged t-test p-values were used as the classification variable. AUC PLIER, AUC RMA and AUC Uniform values were computed. As shown in Table S3, in all the 5 tissues, both the AUC PLIER and AUC RMA values were significantly greater than the AUC Uniform, indicating significant enrichment of probesets with low p-values in the test set. When comparing PLIER and RMA based on the distribution of the AUC values, we found that RMA outperformed PLIER in the cerebellum, and PLIER outperformed RMA in the other four tissues. Of all the 500 classifications (5 tissues times 100 random sampling for each tissue), AUC PLIER values were greater than AUC RMA values in 359 cases (Fisher's exact test p=1.01E-12)

Next, we tested whether the exon inclusion/exclusion patterns detected using the exon array data agreed with those reported in the TISA database. Although the distribution of p-values suggests a level of agreement between the exon array data and the TISA database, we did not find significant consistency in the detected exon inclusion/exclusion patterns. As indicated in Table S2, for the 388 probesets in the test set, for PLIER-summarized data, 206 (53.1%) showed consistent exon

inclusion/exclusion patterns, while for RMA-summarized data, 213 (54.9%) showed a consistent inclusion/exclusion pattern. The percentage of consistent probesets was not significantly greater than 50% (Chi square test p=0.389 and 0.172 for PLIER and RMA, respectively). By focusing only on probesets with significant tissue-specific splicing on the exon array (t-test p<0.05), the percentage of consistent probesets was increased slightly to 56.8% and 60.9%, respectively, but was still not significantly greater than 50% (Chi square test p=0.189 and 0.053), although the p value was close to p=0.05 for RMA. The difference in percentage of consistent probesets between PLIER and RMA was also non-significant (p=0.614 for all probesets and p=0.44 for probesets with t-test p<0.05).

In summary, although both PLIER- and RMA-summarized data showed enrichment of probesets with low p-values in the test set, the exon inclusion/exclusion patterns detected with the exon array data were not generally consistent with those reported in the TISA database, regardless of the summarization method used. PLIER outperformed RMA regarding the enrichment of low p-values in the test set (statistically significant), while RMA outperformed PLIER regarding the consistency in the exon inclusion/exclusion patterns (non-significant). Due to the low level of agreement between the exon array data and the TISA database, we did not reach a conclusion as to which method was better based on these observations.

Reference

[1] Affymetrix white paper: Exon Probeset Annotations and Transcript Cluster Groupings (http://www.affymetrix.com/support/technical/whitepapers/exon_probeset_trans_clust_whitepaper.pdf)

Table S1 AUC values calculated with PLIER and RMA for detection of tissue-specific gene expression

tissue	mean ± std of AUC PLIER	mean ± std of AUC RMA	mean ± std of AUC Uniform	# times of AUC RMA> AUC PLIER (out of 100)	Fisher's exact test PLIER vs. RMA[1]	t-test PLIER vs. RMA[1]
cerebellum	0.777 ± 0.01	0.785 ± 0.01	0.49 ± 0.01	95	1.04E-13	3.24E-8
heart	0.719 ± 0.01	0.73 ± 0.01	0.51 ± 0.01	98	1.33E-16	3.81E-12
kidney	0.757 ± 0.008	0.764 ± 0.007	0.49 ± 0.01	97	1.57E-15	5.95E-10
liver	0.792 ± 0.008	0.787 ± 0.008	0.51 ± 0.01	7	3.45E-12	2.35E-8
muscle	0.719 ± 0.012	0.718 ± 0.012	0.49 ± 0.01	46	0.336	0.307
pancreas	0.582 ± 0.024	0.593 ± 0.022	0.51 ± 0.01	77	5.95E-5	5.65E-4
prostate	0.698 ± 0.01	0.721 ± 0.01	0.49 ± 0.01	100	2.22E-19	9.2E-40
spleen	0.79 ± 0.012	0.814 ± 0.012	0.51 ± 0.01	100	2.22E-19	2.61E-30
testis	0.813 ± 0.007	0.836 ± 0.007	0.49 ± 0.01	100	2.22E-19	1.15E-57
thyroid	0.72 ± 0.019	0.713 ± 0.02	0.51 ± 0.01	20	6.95E-6	0.0183

1 The test is one-sided

Table S2 Number of probesets with consistent exon inclusion/exclusion patterns detected with the exon array and reported in the TISA database.

Tissue	PLIER				RMA			
	# all probesets	# consistent probesets	# probesets with significant AS[1]	# consistent probesets with significant AS[1]	# all probesets	# consistent probesets	# probesets with significant AS[1]	# consistent probesets with significant AS[1]
cerebellum	63	35	28	16	63	25	33	14
heart	17	10	10	6	17	10	9	6
kidney	32	15	13	6	32	15	9	4
liver	53	23	25	14	53	29	20	13
muscle	43	21	25	14	43	24	21	16
pancreas	6	2	1	0	6	1	2	0
prostate	12	4	1	1	12	7	1	1
spleen	23	12	12	4	23	13	6	5
testes	136	83	80	50	136	87	53	35
thyroid	3	1	2	1	3	2	2	1
total	388	206	197	112	388	213	156	95

1 The significant level is $p<0.05$

Table S3 AUC values calculated with PLIER and RMA for detection of tissue-specific splicing

tissue	mean ± std of AUC PLIER	mean ± std of AUC RMA	mean ± std of AUC Uniform	t-test PLIER vs. Uniform[1]	t-test RMA vs. Uniform[1]	# times of AUC RMA> AUC PLIER (out of 100)	t-test PLIER vs. RMA[1]	Fisher exact test PLIER vs. RMA[1]
cerebellum	0.518 ± 0.033	0.626 ± 0.03	0.496 ± 0.052	2.54E-04	8.13E-50	100	6.21E-62	2.23E-19
kidney	0.624 ± 0.043	0.56 ± 0.052	0.508 ± 0.072	9.95E-30	1.37E-8	14	1.24E-18	3.1E-08
liver	0.634 ± 0.036	0.605 ± 0.033	0.496 ± 0.058	1.97E-46	1.44E-35	20	5.64E-09	6.95E-06
muscle	0.756 ± 0.036	0.661 ± 0.034	0.497 ± 0.068	1.01E-72	1.49E-47	2	1.33E-47	1.33E-16
testes	0.652 ± 0.024	0.599 ± 0.021	0.491 ± 0.036	9.00E-86	1.64E-60	5	9.06E-39	1.04E-13

1 The test is one-sided
